# Computers and compassion in general practice

**DOI:** 10.3109/02813432.2013.846545

**Published:** 2013-12

**Authors:** Helena Liira

**Affiliations:** Professor in General Practice, University of Tampere, Finland E-mail: helena.liira@uta.fi

At the Nordic Congress of General Practice in Tampere [1], the largest debate arose after a plenary presentation on information technology for care planning and patient empowerment by Dr Ilkka Kunnamo. Many GPs felt that the visions of the new technologies between doctor and patient were threatening and undesirable while others thought they were intriguing and beneficial. It seemed that there were two parties: GPs who put compassion and communication as the first thing on their agenda and the others who were inspired by the prospects of computer science.

There is nothing new in this debate. In 1967, Alvar Feinstein wrote an article about compassion, computers, and the regulation of clinical technology [2]. Almost 50 years ago this article described computers entering clinical practice, and the themes in it are strikingly similar today. Feinstein wrote that “from the most ancient civilizations to the modern, the recorded attitudes about the ethical consequences of technologic advance indicate that man has been alarmed but assimilating, concerned but consuming”.

While GPs differ in their willingness to adopt new technologies, what about the patients and how would they like their GPs to deal with these matters? Do they prefer computer-savvy GPs or ones that concentrate mostly on good communication? There is little direct evidence to answer these questions. Angela Coulter from the UK has studied patient preferences for a long time. According to her, patients want GPs who are good communicators and have sound, up-to-date clinical knowledge and skills [3]. And perhaps this is in fact the essence: that we must preserve the core values of being good GPs and still introduce technology that supports the development of high-quality medicine.

For up-to-date clinical knowledge, physicians need computerized information resources. Speed and trust are the most important drivers for the selection of sources [4]. Also, patients have become active users of internet-based health information. For GPs it is no longer enough to deliver information to their patients; they also need to discuss the information the patients have found on the internet and guide them to reliable and accurate health websites [5].

Half a century ago Feinstein did not see computers as a threat but as an opportunity. He wrote about computers as mere devices; tools that can help a clinician in providing care and compassion to his or her patients. He also pitied that there were few clinicians active in research for developing and regulating the clinical use of computers.

Studies on information technology as a clinician's device still remain rare. One such example was a study from Norway by Rokstad et al. [6] on electronic optional guidelines. It showed that the quality of referrals was improved by guidelines and that GPs considered electronic guidelines easy to use and time-saving.

It is positive and important that GPs discuss new technologies. All new innovations also include risks that can best be regulated by critical appraisal based on research. Hopefully, this debate will encourage GPs to carry out studies testing new technologies in clinical settings, and their advantages and disadvantages.
